# In the Wards at Great Ormond-Street Hospital for Children

**Published:** 1889-10-05

**Authors:** 


### The Hospital Workshop.

### No. X.—IN THE WARDS AT GREAT ORMOND STREET HOSPITAL FOR CHILDREN.

(By our Special Commissioner.)

At present this building is incomplete, but the foundations are laid of a large additional wing, which will cost £25,000. Great efforts have been made to obtain that sum, and with such success that, whereas four years ago £2,000 only had been raised, the total has since mounted steadily to £16,500. Of this £6,000 represented the Children's Jubilee Tribute, and £4,500 was obtained at special drawing-room meetings, so that the Committee of Management is hopeful of soon reaching the desired limit. Under existing conditions accommodation is cramped, especially that for the nurses, who live in two private houses connected with the main building by subways. When completed the new wing will give room for ninety more beds, and for other necessary improvements.

The hospital is of modern type, airy, light, and comfortable, and is handsome without being needlessly ornate. Under the guidance of the energetic secretary, Mr. Adrian Hope, your commissioner made an interesting tour of the building. The first ward visited was the Victoria, a glance inside which conveys a general impression of homeliness and comfort. There are birds and goldfish, plants and flowers in plenty, pictures line the walls, and toys lie everywhere around. Quite in keeping is the look of the little patients. Some are placidly sleeping in their cots, for the most part ranged down the sides of the room. Others sit up in bed, engrossed in their playthings, while a few are dressed and walking about. Here is a girl of twelve with a stiff neck. The fact that she wears earrings is explained by her nationality. She is the daughter of Jewish parents who used to live in Aldgate, but are now both dead. The poor child is crippled with rheumatism; her knees are affected, and one of her hands, as well as her neck, is swathed in flannel. She tells us she was ill in Norwood Schools for a year, and has been in this place for the last three months. A boy of five walking about the ward is a well-known resident, having been an in-patient no less than three times since his second year. He is one of those curious cases of numerous warty growths in the throat. On one occasion his breathing became so hampered that he was nearly choked, and the throat had to be opened by the operation of tracheotomy. It is doubtful if he will ever be able to dispense with the tube that was then inserted in the windpipe, and through which he breathes. At the end of the ward is the lavatory, its arrangements being excellently carried out in every detail. The box, for instance, is ventilated by the draught of an extraction shaft, heated with a Bünsen's burner. Many of the cots in this and other wards are endowed or kept up from private sources. Among them we noted one supported by the English residents of St. Petersburg, at a cost of £40 a year, and the secretary remarked they are now trying to raise a fund of £1,000 as a permanent endowment. A second is founded in memory of a lost child; a third is "The Mayfair Cot," maintained through church collections by the Rev. Teignmouth Shore; two more are the gift of schools—Ascham and Oundle; another is paid for by the proprietor of a well-known music hall.

Passing into another ward, we see a little curly-headed Saxon, with fair hair and blue eyes. He is not quite two years old, and is suffering from spinal curvature. It is quite clear that he is used to being examined, for he bends forwards at once to show his back. His present illness dates from his second month, when he had an attack supposed to have been diphtheria. The legs are wasted and doubled up, very likely from disease of the spinal cord. There are no toys on his bed, for the simple reason that when put there, he at once throws them out on the floor. These touches of character amongst children are endless, and would furnish good material to an enquiring student of human nature.

In another cot are two red-jacketed mites put together during the afternoon for company's sake, and a quaint little couple they look. One of them is a boy, with some foreign body, supposed to be the glass peardrop of a chandelier, lodged in his throat. It causes him to breathe in a noisy fashion and to speak hoarsely, while a great rumbling can be heard by the stethoscope laid over the windpipe. He coughs sometimes in a peculiar whooping manner, and has undergone the operation of "tracheotomy," but the offending peardrop could not be found. Just now he is playing in a most emphatic way with a model tiger, which he is drumming on his toyboard. Many of the playthings show signs of wear and tear, and the dolls, judging from what we saw on our visit, must require quite a hospital of their own. In the next bed a small boy is sitting with his arms clasped round a highly ornamental birdcage, in which a canary is unconcernedly perched. One day the bird flew into the ward through an open window, was caught, and soon proved to be quite tame and used to children. In answer to the question, "Do you know what bird that is?" the child answered readily, "Yes, Dick." "But what sort of bird is it?" "Dick." "Do you talk to him?" "Yes, Dick." Then the shyness began to wear off and some further details were volunteered. "Dick dinks some water; Dick dinks down dere," along with other facts gathered from a prolonged study of the habits of his pet.

At this point the boy with the peardrop in his throat set up a lusty shouting, and every breath being drawn in with a loud whoop. After a few minutes, however, with all the abruptness of baby grief, he stopped suddenly as he had begun. By the way, this same youth used to have the canary, but it was taken away from him because he would insist on stealing the sugar. This ward is well off for amusements, for there is a fine looking musical box, a rocking horse, goldfish, and a large model of a dun cow with a real "moo." Passing along, we notice that one of the youngsters is making a plaything out of a nurse's chatelaine. Here is a case of cleft palate in a child six and a-half years old, a good time for operation on a healthy subject.

The age is entered upon each patient's card. Here, for instance, we read $1\frac{3}{12}$, a fractional entry that is puzzling for the moment. A little consideration, however, shows that a year and three months must be meant. As a rule, on account of the crying and extra trouble, not more than a couple of infants under two years are allowed in each ward.

A Norfolkshire lad to whom we spoke seemed very anxious there should be no mistake about his age: "Grandmother told them I was eight, but I am really nine." The poor boy has been sadly in the wars. First of all, years ago he had a severe burn on the thigh, and when that was getting better dislocated his hip through a fall. Disease of the joint followed, for which he was treated by a bone-setter, who, in his wisdom, is said to have applied brown sugar and soap to the injuries. The result of this enlightened treatment not being satisfactory, he has been brought to Ormond-street to see if the London surgeons can do anything to straighten his leg, although it is too late to hope for very much improvement. His cot is next the screen from the doorway, and when asked who was in the bed on the other side, he answered "Oh! that little boy. His name's Mosey." "Where does he come from and what is the matter with him?" "I don't know." It seems rather odd that so bright and friendly a child should not have found out all about his next-door neighbour, but he soon gives us the key: "Mosey can't speak. He's a little German; we call him 'German sausage.'"

When asked whether there were any cases of special interest in her ward, the charge sister remarked "Oh, yes; there's our tumour baby." This turned out to be an infant of nine months, the child of a Buckinghamshire gardener. A short time previously, a rapidly growing malignant (*i.e.*, "cancerous" in the popular sense) tumour, the size of an orange, had been removed from the neck. After the operation one side of the face became palsied. The right eyelid drooped and the right pupil was contracted, while the mouth was drawn to the left. Among other points of interest there was sweating of the left half of the face, beginning exactly from the middle line.

The chapel forms a specially interesting feature in this hospital. It lies at the other side of the hall from the main entrance, and was presented to the institution by a gentleman in memory of his wife, all working expenses being defrayed from the same quarter. The architecture is a pure and beautiful example of Byzantine, and the effect on a visitor coming into its quietude from the busy outside world is curiously solemn and suggestive. The architect, the late Mr. Edward Barry, has carried out his work in a style of ornate, yet massive compactness. There is a central dome, supported by marble pillars with alabaster bases and gilded capitals. On either side, an arched recess contains a panel with finely-painted fresco. The altar is cut off by heavy metal rails, and lies beneath a domed apse. The windows are of stained glass, by Clayton and Bell. There is a profuse amount of gilding and decoration, but everything is kept under that kind of artistic command which obliges one to search out beauties of detail. Prayers are held here morning and night for the household; the children only attend service on Saturday afternoon. As a specimen of domestic chapelry the place is well worthy of a visit, and any of the public who wish are at liberty to attend the short service which is held every Saturday at 4.45 p.m. The chaplain belongs to the Established Church, but the hospital, in accordance with a well-recognised rule, does not profess to have any religious denomination. The cost of this building must have been considerable, and whatever opinion may be held as to the wisdom of this kind of outlay on a charitable institution, there can be no doubt whatever as to the spirit which has prompted the founder, nor as to the perfect and solemn beauty of the monument.

